# Placental methylome analysis from a prospective autism study

**DOI:** 10.1186/s13229-016-0114-8

**Published:** 2016-12-15

**Authors:** Diane I. Schroeder, Rebecca J. Schmidt, Florence K. Crary-Dooley, Cheryl K. Walker, Sally Ozonoff, Daniel J. Tancredi, Irva Hertz-Picciotto, Janine M. LaSalle

**Affiliations:** 1 Department of Medical Microbiology and Immunology, Genome Center, Davis, CA 95616 USA; 2 Department of Public Health Sciences, University of California, Davis, CA 95616 USA; 3 Department of Obstetrics and Gynecology, University of California, Davis, CA 95616 USA; 4 Department of Psychiatry and Behavioral Sciences, University of California, Davis, CA 95616 USA; 5 Department of Pediatrics, University of California, Davis, CA 95616 USA; 6 MIND Institute, University of California, Davis, CA 95616 USA

**Keywords:** Epigenetics, Genomics, DNA methylation, Methylome, Placenta, Biomarkers

## Abstract

**Background:**

Autism spectrum disorders (ASD) are increasingly prevalent neurodevelopmental disorders that are behaviorally diagnosed in early childhood. Most ASD cases likely arise from a complex mixture of genetic and environmental factors, an interface where the epigenetic marks of DNA methylation may be useful as risk biomarkers. The placenta is a potentially useful surrogate tissue characterized by a methylation pattern of partially methylated domains (PMDs) and highly methylated domains (HMDs) reflective of methylation patterns observed in the early embryo.

**Methods:**

In this study, we investigated human term placentas from the MARBLES (Markers of Autism Risk in Babies: Learning Early Signs) prospective study by whole genome bisulfite sequencing. We also examined the utility of PMD/HMDs in detecting methylation differences consistent with ASD diagnosis at age three.

**Results:**

We found that while human placental methylomes have highly reproducible PMD and HMD locations, there is a greater variation between individuals in methylation levels over PMDs than HMDs due to both sampling and individual variability. In a comparison of methylation differences in placental samples from 24 ASD and 23 typically developing (TD) children, a HMD containing a putative fetal brain enhancer near *DLL1* was found to reach genome-wide significance and was validated for significantly higher methylation in ASD by pyrosequencing.

**Conclusions:**

These results suggest that the placenta could be an informative surrogate tissue for predictive ASD biomarkers in high-risk families.

**Electronic supplementary material:**

The online version of this article (doi:10.1186/s13229-016-0114-8) contains supplementary material, which is available to authorized users.

## Background

Autism spectrum disorders (ASD) are currently estimated to affect one in 68 births in the USA [1]. Diagnosis of ASD typically occurs in children 3 years old or later through the Autism Diagnostic Observation Schedule (ADOS) that identifies impairments in social interaction and communication, as well as restrictive and repetitive interests and behaviors [2, 3]. Having an older sibling with ASD increases the risk for ASD, especially if multiple older siblings are affected [4]. Research into genetic causes of ASD has been extensive and has identified multiple pathogenic mutations and copy number variants (CNV) [5, 6]. However, any single genetic cause makes up <1% of total ASD cases, and the majority of ASD cases appears to be multifactorial, involving complex interactions between genetic and environmental risks and protective factors [7]. Epidemiological evidence suggests that periconception and in utero periods are the most vulnerable to environmental factors influencing ASD risk [8–12]. Since early identification and behavioral intervention in ASD has improved outcomes in individuals with ASD [13], an important goal is to develop molecular biomarkers that could predict increased ASD risk at birth.

Epigenetic marks such as DNA methylation are at the interface of genetic and environmental risk and protective factors in ASDs and therefore could make ideal biomarkers [14]. However, choice of a surrogate tissue is critical for epigenome-wide association studies since the brain is not accessible and blood DNA methylation patterns are influenced by variables such as cell type heterogeneity [15]. Also, blood cells have cell lineage differences from neurons that may impact the ability to detect methylation differences relevant to the brain. The placenta is a readily accessible tissue at birth that is normally discarded but could offer a unique epigenetic window into the interface of genetic and environmental factors that were present in utero.

The human placenta has a distinct methylation landscape found throughout all the three trimesters of pregnancy, characterized by large partially methylated domains (PMDs) interspersed with highly methylated domains (HMDs) [16]. PMDs are usually over 100 kb in length and cover tissue-specific, transcriptionally repressed genes [17]. Interestingly, neuronal development and synaptic transmission genes are statistically overrepresented in the placental PMDs, as are autism candidate genes [17]. The large-size and regionally defined methylation levels of PMDs make them amenable to analysis in low coverage (×1–2) whole genome bisulfite sequencing datasets [16, 18], which are much more affordable to generate for clinical samples than individual CpG resolution analysis at ×30 coverage. Our prior study demonstrated that high versus low coverage placental MethylC-seq analyses show nearly identical global methylation patterns, with pairwise correlations of >0.95 [18].

Because hypomethylation in the placenta probably derives from the hypomethylated state of the early embryo and trophectoderm, disturbances in the large-scale methylation patterns of the placenta could be indicative of methylation irregularities present in the embryo, which could later affect neuronal development in the fetus [18]. Placental inclusions, which are markers for genetic abnormalities and abnormal trophoblast infoldings, were previously observed in increased numbers in placental samples from participants in MARBLES (Markers of Autism Risk in Babies: Learning Early Signs), who are at high risk for developing autism compared to a general clinical population sample [19]. In this study, we performed whole genome methylome analyses on MARBLES placental samples to determine the utility of placental samples in identifying methylation markers indicative of ASD risk.

## Methods

### MARBLES study and sample selection

The placental tissues were obtained as previously described [19] from the MARBLES (Markers of Autism Risk in Babies: Learning Early Signs) study, a prospective study of the environmental, genetic, and epigenetic factors leading to autism spectrum disorder (ASD) (Hertz-Picciotto, in revision). Pregnant women and women planning a pregnancy were enrolled if they or the father already had a child with ASD; they were thus at significantly (13-fold) higher risk of having another child with ASD, as compared with parents not having a previous child with ASD [4]. MARBLES recruited Northern Californian families from lists of children receiving services for ASD funded through the California Department of Developmental Services. Proband ASD status was confirmed, and the mothers were seen at regular intervals during pregnancy with biosample collection starting at enrollment and repeated at each visit. The placenta, cord blood, and other samples were collected and frozen at birth, and the children were followed to age 36 months when a final developmental diagnosis of autism or typical development was made by ADI-R in addition to the other assessments. DNA was isolated for MethylC-seq analysis from MARBLES placentas from births of 24 children who received a final 36-month clinical diagnosis of ASD by September 29, 2014 and 23 typically developing children matched to the children with ASD by gender and birth year (within 1.5 years), with preference given to those with greater availability of other study data. Due to the naturally occurring high proportion of males to females with autism, seen also in the MARBLES study, only two in each of the autism and typical placentas came from females (Additional file 1: Table S1). For non-MARBLES population control samples, full-term human placental samples were obtained from routine Cesarean sections, as described previously [16]. All participants gave written informed consent for data and sample use, and protocols were approved by UC Davis IRB (protocol # 225645-35).

### Diagnostic classification

Children’s development was assessed by trained and reliable examiners with final diagnostic assessments at 36 months. All children are assessed for autism symptoms using the gold standard Autism Diagnostic Observation Schedule-Generic (ADOS-G) [20, 21]. A clinical best estimate diagnosis was given through the consensus of two clinicians based on *DSM-IV* or *DSM-5* criteria. Placental samples were categorized based on whether the child met ADOS and *DSM* criteria for ASD, showed typical development, (TD), or had impairments in some domains, but did not meet the full ASD diagnostic criteria (ODC for Other Developmental Concerns).

### MethylC-seq

The placental samples were frozen immediately after birth. DNA was extracted using Qiagen’s Puregene kit, sonicated to ~300 bp, and methylated Illumina adapters were ligated to the ends using NEB’s NEBNext DNA library prep kit. The library was bisulfite-converted using Zymo’s EZ DNA Methylation-Lightning Kit, amplified for 14 cycles using PfuTurbo Cx, purified with Agencourt AMPure XP beads, and sequenced on an Illumina HiSeq 2000. Reads were mapped to the hg19 version of the human genome using BS Seeker [22]. To eliminate clonal PCR amplification duplicates, only one read out of those with identical genomic positions was kept; Genome and CpG coverage was estimated by multiplying the number and the length of the mapped reads and dividing by the size of the human genome (Additional file 1: Table S1). CpG site methylation data were combined from both DNA strands.

### Defining PMD/HMDs

Methylation data from 17 typical placentas from MARBLES were combined to create a single, high-coverage map of methylation across the genome. Visually annotated PMD and HMD portions of this consensus genome were used to train a two-state hidden Markov model (HMM) to differentiate PMDs and HMDs using an HMM called hidden Markov models of methylation (StochHMM) [23], as previously described [16]. The model was then applied to the same high-coverage methylation data to define the boundaries of PMD/HMDs in the typical placentas. Those boundary chromosome coordinates were used for calculating average percent methylation in both typical and autism placental samples in the MARBLES study. For each sample, the average % methylation over all PMDs and all HMDs was calculated. In addition, % methylation for each PMD and HMD for each individual was calculated, and differences between ASD and TD samples were assessed for significance using two-tailed *t* tests and false discovery rate (FDR) multiple hypothesis correction (0.05).

### Determination of maternal blood contamination by X chromosome methylation

Since the majority of fetal samples were male that only contains a single active X chromosome, we used DNA methylation values from specific regions of the X chromosome that are specifically methylated in females due to X chromosome inactivation on the second X chromosome. All CpG islands within HMDs on the X chromosome were selected, and the mean percent methylation was determined. Since a sample containing no female cells would theoretically be expected to have lower methylation over these regions, the percentage of female cells in each sample was estimated and compared between samples for potential differences.

### Methylation pyrosequencing

Samples were taken from six different locations in ten full-term control placentas (non-MARBLES samples and primers described previously [16]. Genomic DNA was isolated and bisulfite-converted as above, PCR-amplified for 45 cycles using HotStarTaq polymerase (PyroMark PCR Kit, Qiagen), and run on a PyroMark Q24. Primer sequences for the DLL1 locus were from hg19 chr6:170,534,692-170,534,733 spanning three CpG sites:

- DLL_enh_BS_F1:TTGGGTTTAGTTGGGGATAGGG
- DLL_enh_BS_R1:AACCCAAAAACTTCCCTCTC
- DLL_enh_BS_S1:TTTATTTGTTTGTTATAGTTTGAG

### Statistical analyses for associations between methylation and sequencing and demographic factors

Information on demographic factors were collected through telephone-assisted interviews. The following variables were analyzed for associations with PMD total average methylation, HMD total average methylation, and the percent of the 20 kb windows with methylation below 60%: sequencing run, order, and coverage; child race (white non-Hispanic [reference], Asian, multi-racial, white Hispanic, non-white Hispanic); and child sex (male [reference]/female).

We performed univariate linear regression using the SAS software version 9.4 for each variable in relation to PMD methylation, PMD methylation adjusted for HMD methylation, HMD methylation, HMD methylation adjusted for PMD methylation, and the percent of the 20 kb windows with methylation below 60% (using a 5% change as the unit) (Additional file 1: Table S6). To facilitate interpretation of regression coefficients, PMD and HMD methylation was expressed in 5 percentage point and 2 percentage point units, respectively, so that coefficients (and effect size estimates based on them) correspond to an approximately 2 SD change in the independent variable. To account for the multiplicity of hypotheses being assessed, we controlled the FDR at 5% [24]. In ad hoc analyses, we examined demographic factors in relation to *DLL1* methylation (unit was 5% change). We used the Akaike information criterion (AIC) to select the most parsimonious among the candidate models [25, 26]. To produce a final model, we used sandwich estimators to produce homoscadisticity-robust estimates of the 95% confidence interval given that the conditional variance of outcomes appeared to vary with the regression-based predicted values.

## Results

### Human placentas have highly reproducible PMD/HMD patterns with higher variability over PMDs than HMDs

**Fig. 1:**
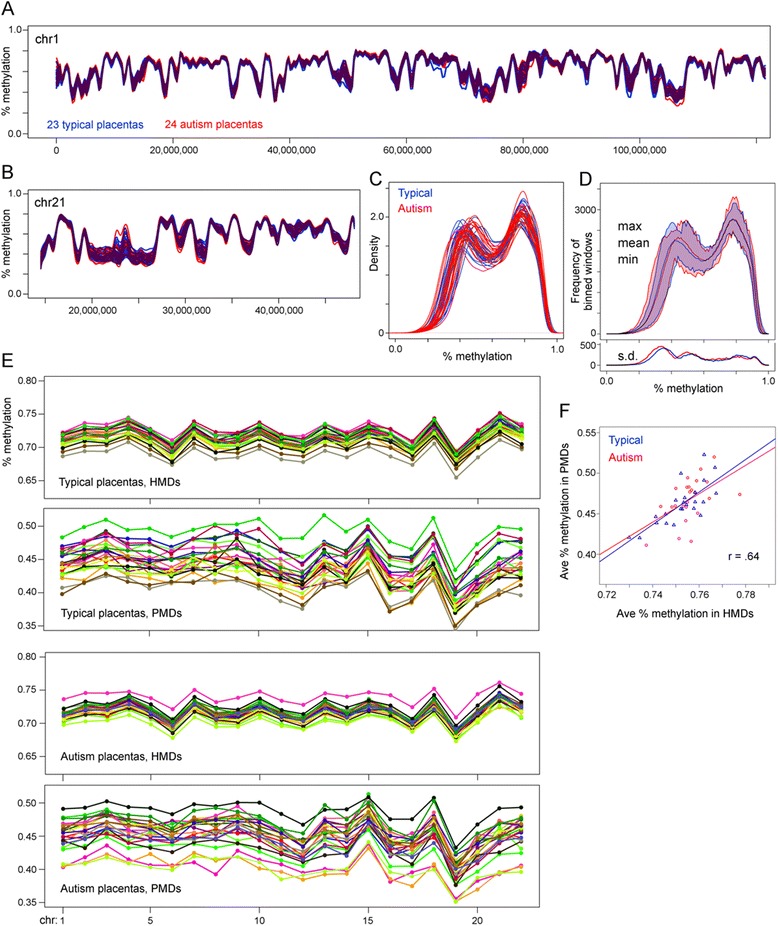
Genome-wide methylation landscape of MARBLES typical and autism placentas. **a**–**d** Average methylation in non-overlapping 20 kb windows tiled across the autosomes. CpG islands were not removed. **a** Methylation across the p arm of chromosome 1 and **b** the q arm of chromosome 21. *Curves* were smoothed with a first order local polynomial. Overlap between autism and typical is shown in *maroon*. **c** Distribution of average window methylation across all autosomes with kernel density smoothing. **d** Because of high curve overlap in **c**, the average window methylation was binned at 1% methylation increments for each sample, and the minimum, mean, maximum, and standard deviation were plotted for both typical and autism placentas. No additional smoothing was done. **e** Average methylation of CpG sites in PMDs/HMDs on each individual autosome. PMD/HMD boundaries were determined using a hidden Markov model on a combined set of 17 typical placentas. Each individual placenta has the same color in the HMD and PMD graphs. **f** Correlation between genome-wide methylation levels in PMDs and HMDs for each placenta. Least squares regression lines are shown for the typical and autism placentas. The Pearson correlation for all samples combined is 0.64

MethylC-seq analysis of 24 ASD and 23 TD placental samples showed that global genome-wide methylation patterns were very similar between individuals, with methylation variability between samples being larger within the PMDs than HMDs (Fig. 1a, b, Additional file 1: Table S1). In particular, there seemed to be a little deviation in the locations of PMD/HMDs and their boundaries. When graphing average methylation in 20 kb windows across the genome, PMD/HMDs show up as a bimodal distribution in the human placenta [16]. Similar bimodal curves were seen in all typical and autism MARBLES placentas (Fig. 1c, d, Additional file 2: Figure S1). Greater inter-individual variability was observed in PMDs as a higher standard deviation over the peaks with lower methylation (Fig. 1d).

Since there is some difference in methylation between individuals, particularly in PMDs, we next asked if higher versus lower average methylation for a given individual (relative to the group) is consistent across chromosomes or different between chromosomal loci. In Fig. 1a, b, individual samples appeared follow a similar pattern across each chromosome, and that pattern was also observed for each sample graphed by average methylation for each chromosome for each individual (Fig. 1e). Individuals with higher or lower methylation in HMDs on one chromosome also tended to have similar higher or lower methylation in HMDs on the other chromosomes. Genome-wide methylation levels in HMDs varied by as much as 4.76% between individuals. For PMDs, the same trend of consistently higher or lower methylation across chromosomes was still observed, but there was a more random mixing of relative methylation levels in PMDs compared to HMDs. Genome-wide methylation levels in PMDs varied by as much as 11.17% between individuals. These genomic individual differences in methylation levels between individuals were observed in both autism and typical placental samples, with negligible differences between diagnostic groups.

Note that in Fig. 1e, a typical placenta was given the same color in both the PMD and HMD graphs (likewise for the autism placentas). Since individuals with relatively high or low methylation in HMDs did not necessarily have the same relatively high or low methylation in PMDs, we next asked how well genome-wide average methylation levels in PMDs and HMDs correlated. For both typical placentas and autism placentas, there are significant positive correlations between methylation levels in PMDs and HMDs, with a combined correlation of 0.64 (Fig. 1f).

### Intra-tissue DNA methylation variability mirrors inter-individual variability

**Fig. 2:**
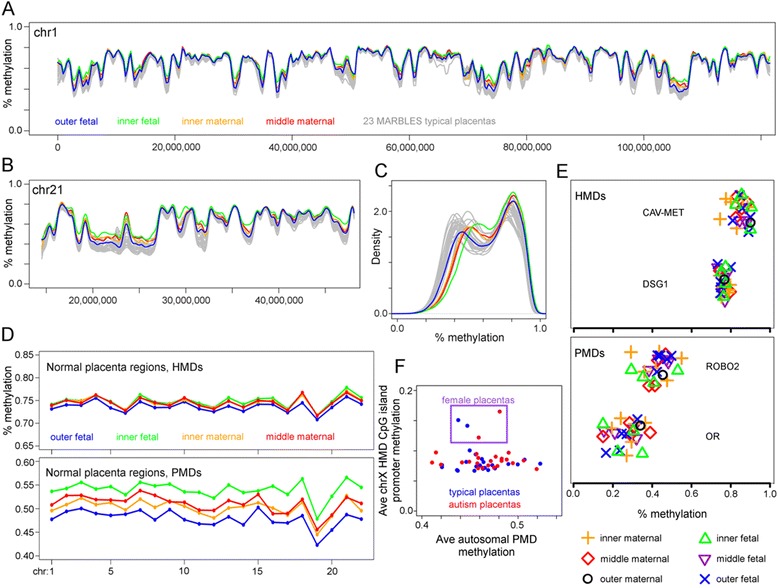
Genome-wide methylation analysis of tissue heterogeneity within the human placenta. **a**–**c** Four samples were taken from the maternal to the fetal side of a single full-term human placenta. The term placenta was not from the MARBLES study and had no known pathologies. Average methylation was calculated in non-overlapping 20 kb windows tiled across the autosomes. CpG islands were not removed. For comparison, the 24 MARBLES typical placental samples are shown in *gray*. **a** Methylation across the p arm of chromosome 1 and **b** the q arm of chromosome 21 as examples. *Curves* were smoothed with a first order local polynomial. **c** Distribution of average window methylation across all autosomes with kernel density smoothing. **d** Average methylation of CpG sites in PMDs/HMDs on each individual autosome. PMD/HMD boundaries were determined as in Fig. 1e. **e** Blinded methylation pyrosequencing of two PMDs and HMDs from 24 placental samples. Samples were randomly selected from six placental regions in ten non-MARBLES placentas with no known pathologies. Data were jittered along the *y* axis for visibility. Data from Schroeder et al. (2013). **f** Effect of maternal blood contamination on PMD methylation in male placentas. To epigenetically quantify maternal blood contamination levels, DNA methylation was assessed in the CpG island promoters of genes in chrX HMDs. These regions normally have low methylation in males but higher methylation in females due to X chromosome inactivation

Although PMD/HMD patterns were consistent between individual placentas, we next asked how much of the variability in the relative level of methylation between individuals could be due to tissue sampling heterogeneity. More specifically, are methylation levels or patterns different on the maternal versus fetal sides or between the different regions within the placenta? To answer this question, we sampled the outer fetal, inner fetal, inner maternal, and middle maternal portions of a single normal placenta that was not part of the MARBLES study. Figure 2a–d shows that the variability in relative methylation levels between regions within a single placenta are similar to the inter-individual variability seen in typical MARBLES placentas. Specifically, the ~10% range of methylation values for PMD intra-tissue variability shown in Fig. 2d is no greater than the PMD inter-individual variability shown in Fig. 1e.

To examine whether placental sampling location was predictive of methylation level, pyrosequencing analysis was performed over four genomic loci, representing 2 HMD and 2 PMD loci described previously [16]. Samples were from ten normal placentas that were not part of the MARBLES study, and each placenta was sampled from one to five of the six placental regions (Fig. 2e). Samples from the same placental region but different placentas did not co-cluster but appeared fairly randomly distributed relative to the other samples. At all four loci, two-way ANOVA tests showed neither statistically significant effects of individual placenta nor placental location on percent methylation over these loci.

We next asked whether the inter-individual variability in methylation observed in PMDs could be attributed to differences in levels of maternal blood contamination. Because global methylation levels in the blood are typically high, similar to other adult somatic tissues, the amount of blood in the placental sample would proportionally raise the amount of observed methylation in PMDs. To address this in the MARBLES placentas, we utilized the epigenetic feature that females have higher methylation in CpG island promoters on the inactive X chromosome [27]. We reasoned that if maternal cells were contributing to placental methylation levels, higher levels of maternal cells in the placentas of male offspring would correspond to increased methylation in X chromosome CpG island promoters. Figure 2f, however, shows that there is no correlation between autosomal PMD methylation and X chromosome CpG island promoter methylation. However, four of the MARBLES samples were from female offspring (marked with a purple box), and increased methylation is observed in their X chromosome CpG island promoters, as would be expected from a female genome.

### Methylation differences between typical and autism placentas

In order to determine if DNA methylation in placenta could be used as a biomarker for early autism detection, we first performed linear regression tests on both global average HMD methylation and global average PMD methylation, looking at the effects not only of child diagnosis (autism or typical) but also sex, the sequencing run number, the order of sequencing (since Illumina sequencing chemistries changed during the course of the study), and the average sequencing coverage. None of the factors, including diagnosis, had statistically significant effects on either average HMD or PMD methylation (data in Additional file 1: Table S2). In addition, no significant difference in percent methylation between ASD and TD samples was observed over any chromosome (data in Additional file 1: Table S3). Average methylation in individual promoters, CpG islands, gene bodies, and non-overlapping 20 kb windows were also tested as above, but no statistically significant differences between ASD and TD samples were found after FDR correction.

**Fig. 3:**
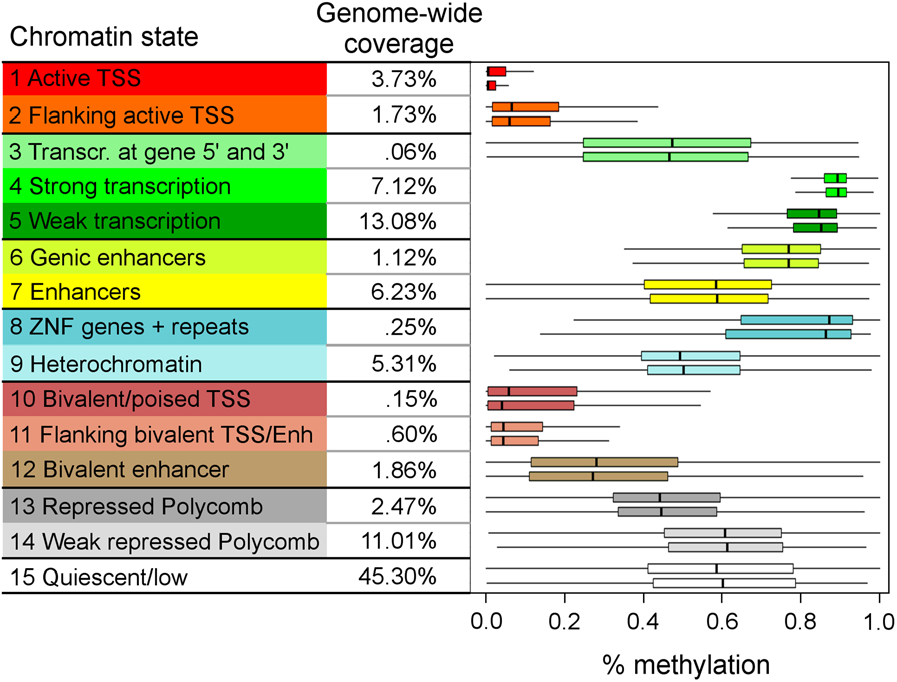
Chromatin states and DNA methylation. Box plots of the distribution of methylation in ChromHMM state elements [28], with each point representing one instance of that ChromHMM state in the genome. For each placental ChromHMM state, percent methylation was calculated for each instance of that state in the genome by averaging over the CpG sites in the element and then averaging over all typical (*top box*) or autism (*bottom box*) placental samples. In the box plots, the *middle dark vertical line* is the median, the *ends* of the box are the interquartile range, and the *ends* of the whiskers extend to the point within 1.5 times the interquartile range. For easier viewing, outliers were not plotted

To determine if there were methylation differences within chromatin states, hidden Markov models of chromatin states (ChromHMM) mappings for the human placenta were investigated [28]. Figure 3 shows the average methylation, across autism/typical placental samples, for each chromatin state. This shows, for example, not only the average methylation for heterochromatin but also the range of methylation found for individual heterochromatin elements. Within the human placenta, both active and poised transcription start sites (TSSs) show low methylation; however, active TSSs show the lowest methylation levels. Transcribed regions show the highest methylation and heterochromatin, Polycomb-regulated, quiescent, and many enhancer regions show partial methylation. In order to determine if ASD and typical placentas differed in their global levels of methylation in any of the chromatin states, average genome-wide methylation for each chromatin state was calculated for each MARBLES placental sample. Two-tailed *t* tests showed no statistically significant differences between ASD and TD samples for any of the ChromHMM states after FDR correction (Additional file 1: Table S4).

**Fig. 4:**
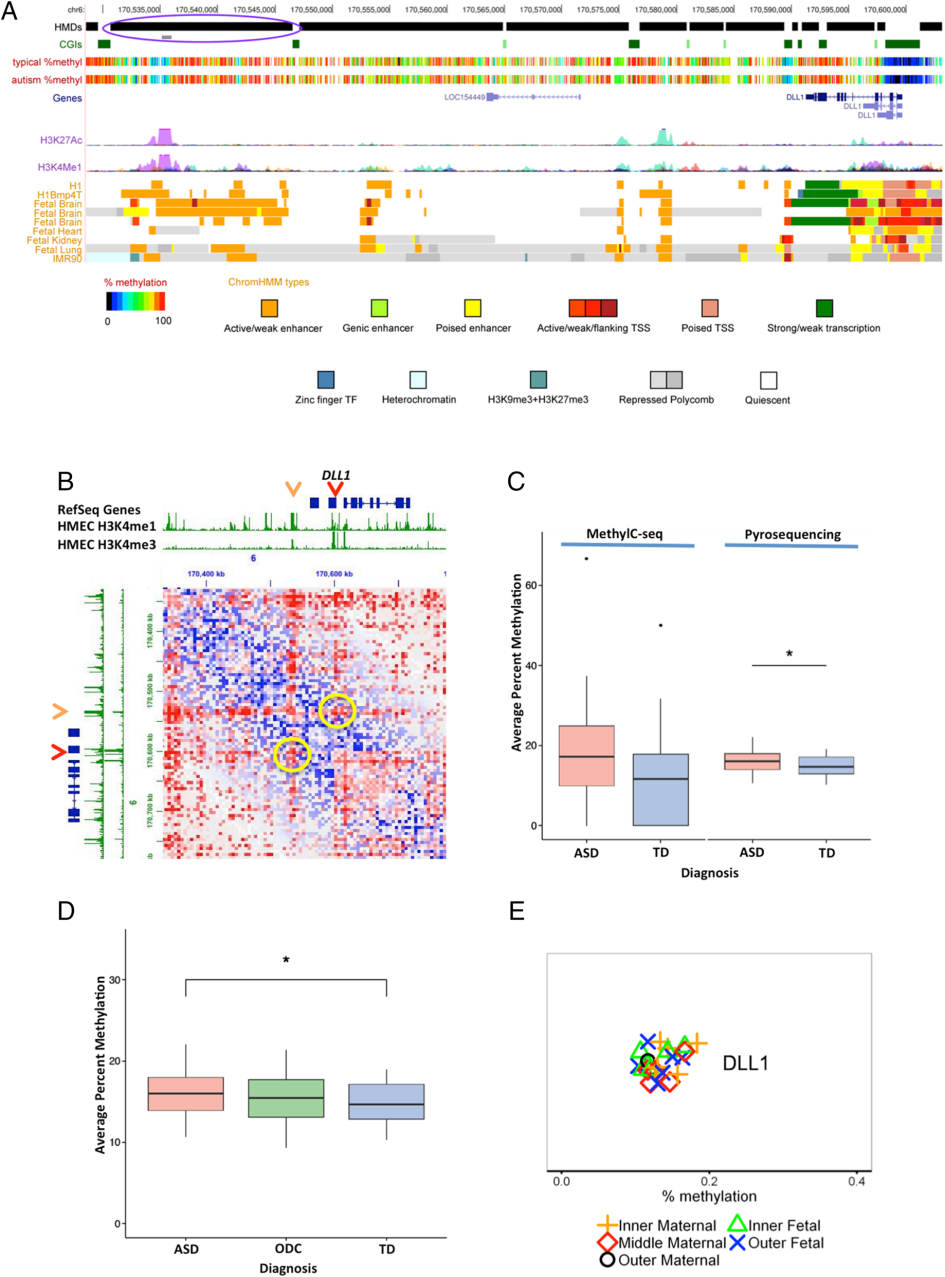
Epigenetic characteristics of differentially methylated region over a predicted enhancer near the *DLL1* gene and validation by pyrosequencing. **a** UCSC Genome Browser screenshot showing the differentially methylated HMD region (*circled in blue-purple*). Methylation data for the 23 typical MARBLES placentas and 24 autism MARBLES placentas were combined to produce composite methylation tracks with each vertical bar showing percent methylation at individual CpG sites. H3K27ac and H3K4me1 tracks are from ENCODE. ChromHMM tracks are from the Roadmap Epigenomics Consortium [28]. *HMD* highly methylated domain, *CGI* CpG island, *H1Bmp4T* H1 human embryonic stem cells differentiated into trophoblasts, *IMR90* human fetal lung fibroblasts, *TSS* transcription start site, *TF* transcription factor. **b** Long-range interactions in the *DLL1* locus revealed by “Juicebox” visualization tool of human HUMEC HiC data (HUMEC in situ combined, observed, *red*; expected, *blue*) [29]. *Red arrow* designates *DLL1* promoter; *orange arrow*, putative *DLL1* enhancer; *yellow circles* highlight evidence for observed 3D interactions over expected for these two loci. **c** Three CpG sites corresponding to the H3K27ac/H3K4me1 peaks were assayed by pyrosequencing on the same samples analyzed by MethylC-seq (region shown by *gray bar* in **a** within *circled* HMD). Average MethylC-seq % methylation for the three CpG sites is plotted compared to the pyrosequencing values. ASD and TD pyrosequencing values from three replicates per sample were tested for significance by *t* test. *p < 0.05. **d** An “Other Developmental Concerns” (ODC) group that was neither TD nor ASD was compared to TD and ASD by pyrosequencing as in **c**. No significant differences were observed between ODC and ASD or ODC and TD, although the values were between the two groups, as expected. *N* = 24 ASD, 23 TD, 23 ODC. **e** *DLL1* pyrosequencing analysis of methylation levels by placental region. Results shown are from six different control placentas and samples obtained from five different regions as labeled

We next tested average methylation in individual PMDs and HMDs using two-tailed *t* tests and FDR multiple hypothesis correction (0.05). No PMDs showed statistically significant differences between autism and typical placental sample; however, one HMD showed significance with a *p* value of 0.0267 (Additional file 2: Figure S2). It is located on chromosome 6, 45 kb downstream from the *DLL1* gene and 16 kb downstream of the *LOC15444* non-coding RNA. This differentially methylated HMD includes a region with the enhancer marks H3K4me1 and H3K27ac in the fetal brain (Fig. 4a). Evidence for three-dimensional interaction between this putative enhancer and the *DLL1* promoter is provided by visualization of this locus in published HiC data (Fig. 4b) [29]. A pyrosequencing analysis of bisulfite-converted DNA using primers designed to the enhancer region validated significantly increased DNA methylation in ASD compared to TD samples (Fig. 4c). In contrast, samples from the “other developmental concerns” (ODC) group in MARBLES with behavioral features that did not fall into either TD or ASD categories showed intermediate methylation levels at this *DLL1* locus (Fig. 4d, Additional file 1: Table S5). Similar to the other specific loci shown in Fig. 2e, methylation analysis of the *DLL1* locus by pyrosequencing did not reveal detectable differences based on location of the placental sample (Fig. 4e).

### Influence of sequencing and demographic factors

Sequencing run, order, and coverage and child race/ethnicity and sex were evaluated as predictors of average PMD and HMD values, as well as the percentage of 20 kb windows less than 60% methylation, in univariate linear regression analysis. None of these factors was a significant predictor of methylation though child race/ethnicity showed a trend toward an association with PMD methylation prior to FDR correction (Additional file 1: Table S6). Sequencing and demographic variables were not independently significantly associated with *DLL1* locus methylation, and the most parsimonious model selected by AIC included only ASD status, which was associated with higher average *DLL1* methylation (estimate = 0.24, 95% CI −0.04, 0.51, *p* = 0.09).

## Discussion

Identifying methylation signatures of risk for neurodevelopmental disorders such as ASD in placenta is a challenging goal that we sought to address with an initial study on the feasibility of using MethylC-seq in placental samples from a prospective ASD study. Despite the inherent limitations in the study design (low coverage of individual CpG sites, small sample size, sampling heterogeneity), several novel findings were obtained by this approach.

First, MethylC-seq and PMD/HMD analyses were successfully used to identify a novel differentially methylated region between ASD and TD placentas corresponding to an apparent fetal brain enhancer near the *DLL1* locus. Differential methylation at this locus was not explained by differences in sequencing or demographic factors between ASD and TD placentas. *DLL1* encodes the *Delta*-like1 ligand of Notch receptors that mediates lateral inhibition of neighboring cells in embryonic development through Hes1 transcriptional feedback. In mouse embryonic brain, Dll1 and Hes1 proteins show reciprocal oscillations in neural precursor cells [30], and *Dll1* oscillations are predicted to act to control proliferation versus differentiation of neurons [31], a developmental period of importance to ASD [32, 33]. Furthermore, loss-of-function mutations have been observed in *DLL1* in human ASD, as well as other members of Notch signal transduction [34]. While this locus has the histone marks and chromatin organization associated with being a strong fetal brain enhancer, future analyses in animal models would be needed to determine the functional relevance of methylation at this epigenomically defined enhancer to *DLL1* expression in the embryonic brain.

A sex hormone imbalance during pregnancy has been implicated to explain the male bias of ASD [35]. In rodents, inhibition of DNA methyltransferases in the sexually dimorphic preoptic brain region resulted in masculinized reproductive behaviors [36]. Furthermore, in human prostate cells, dynamic changes in DNA methylation at regulatory regions corresponded with transcriptional changes in response to androgen treatment [37]. Since Notch signaling and *Dll1* expression are responsive to progesterone in mouse models [38, 39], and human pregnancies resulting in ASD diagnosis showed increased fetal steroidogenic activities from amniotic samples [40], perhaps the higher methylation levels for the putative *DLL1* enhancer observed in ASD versus TD in our study reflect fetal steroidogenic alterations. Future human studies could attempt to detect steroid protein levels in relation to DNA methylation in stored frozen placental samples [41] from high-risk ASD cohorts.

Interestingly, this putative *DLL1* enhancer locus was not represented on the Illumina Infinium 450 k array platform, so prior ASD studies of differential methylation in the brain [42, 43] or in surrogate tissues [44, 45] would not have been able to detect it. Since the current cost for MethylC-seq at the coverage we performed in this study is becoming closer to that of array-based technologies, our approach represents an alternative method with increased genomic coverage for finding epigenetic biomarkers. While transcriptome differences are often used for biomarker discovery, RNA quality is notoriously poor due to nuclease activity of placenta, and the term placenta may not be ideal for uncovering gene expression differences that occurred earlier in gestation. Due to the low coverage of individual CpGs inherent in our approach, however, some relevant methylation differences may have been missed, but this limitation is expected to improve in future studies using whole genome methylation sequencing. Another limitation in our study was the small sample size of currently available placental samples with ASD diagnoses, which may decrease the sensitivity to detect methylation differences in the *DLL1* locus that were due to ASD as opposed to other confounding factors. Small effect sizes for methylation differences are a common finding in children’s studies, but combining multiple putative methylation biomarkers could increase sensitivity of these assays [46]. Prior methylation studies in ASD have identified oxytocin receptor (*OXTR*), Engrailed 2 (*EN2*), and methyl CpG binding protein 2 (*MECP2*) with the largest effect sizes in the brain or blood [14, 47–52]. With additional power from increased sample size in future studies, these and other ASD candidate epigenetic biomarkers may be confirmed or identified in placenta.

In addition, we investigated sources of inter-individual variability in methylation patterns in human placental samples independent of ASD diagnosis. While PMDs are the most interesting epigenetic feature of the placental methylome, these regions are also the most variable between individuals, a potential confounding factor in the search for disease or exposure relevant biomarkers within PMDs. Interestingly, the inter-individual methylation levels appeared to be genome-wide rather than locus-specific, with individual samples showing relatively higher or lower methylation over both PMDs and HMDs. One explanation for variability over PMDs was heterogeneity in sampling location, likely due to the different mixture of cell types represented in different placental regions. At individual PMD loci measured by pyrosequencing, however, sampling location did not apparently account for significant differences. Maternal blood contamination was determined to be less than 10% of cells by methylation analysis of promoters on the X chromosome in male samples, and degree of X-linked methylation did not correlate with average methylation over PMDs, suggesting that this is not a likely source of inter-individual variation in methylation levels over PMDs.

Placental tissue contains a heterogeneous mixture of different cell types, including trophoblasts (cytotrophoblasts and syncytotrophoblasts), mesenchymal stromal cells (fibroblasts and mesenchymal-derived macrophages), fetal vascular cells (smooth muscle cells, pericytes, endothelial cells), and fetal hematopoietic cells (extravascular fetal red blood cells, hematopoietic stem cells) [53, 54]. Therefore, different ratios of these mixed populations of cell types between individual placental samples could be a source of the inter-individual variation observed over PMDs or possible intra-tissue variability not detectable in our analyses. However, a prior comparison between isolated trophoblast cells and the whole placenta in rhesus macaque showed strong correlation between their methylation levels (0.89), suggesting that cell type methylation differences in the placenta may be lower than would be expected [18]. In the cord blood samples, fetal nucleated red blood cells (nRBCs) are hypomethylated relative to other blood cell types, and variable numbers of these nucleated RBCs can affect methylation levels [55]. The possibility that differences in fetal nRBCs could explain inter-individual variation over placental PMDs may be investigated in future studies through cell sorting and data normalization approaches described for cord blood [56].

## Conclusions

In conclusion, whole genome bisulfite sequencing analyses of human placental samples are expected to be useful in the future for the detection of disease methylation biomarkers in prospective studies of ASD with increased sample size.

## Additional files

Additional file 1: Tables S1-S6.
**Table S1**. MethylC-seq sequencing statistics. **Table S2.** Average methylation over all PMDs and HMDs in placental samples. **Table S3.** Average DNA methylation for all chromosomes in placental samples. Table also shows average methylation over all autosomes, which is the average methylation over all CpG sites in the autosomes, not just the average over the chromosome-wide averages. **Table S4.** Average autosomal DNA methylation for ChromHMM states in placental samples. For each ChromHMM state and each placental sample, average methylation was calculated for all autosomal CpG sites in elements of that state. **Table S5.** Pyrosequencing analysis at the *DLL1* locus. Percent methylation for each of the 3 CpG sites (P1, P2, P3) was determined by pyrosequencing for each ASD, TD, and ODC placental sample. **Table S6.** Demographic and environmental factors in relation to placental methylation. Statistical analyses of potential covariates including, run, order, coverage, ethnicity, and sex with methylation of PMD, HMD, % 20 kb windows <60% methylation, and *DLL1* locus. (XLSX 51 kb)
Additional file 2: Figures S1-S3.
**Figure 1.** Histograms of average methylation in 20 kb windows for each placental sample. Individual histograms (no smoothing) for each MARBLES typical and autism placentas as well as three biological replicates of “normal” non-MARBLES placentas which have no known pathologies [16]. Methylation data was omitted if a window had fewer than 20 covered CpG sites. Red vertical lines show the arbitrary 60% methylation cutoff between windows with low and high methylation. Percentage of 20 kb windows with average methylation below 60% also shown in red (excluding omitted windows). *n* = number of windows with required CpG coverage, *m* = number of windows omitted due to insufficient coverage. **Figure S2.** Plots showing location and statistical significance of differential methylation in HMDs and PMDs. Each point shows the FDR-corrected probability of that HMD/PMD being differentially methylated between autism and typical MARBLES placentas. Chromosomes are alternately colored red and black for clarity. The blue line shows the 0.05 cutoff for statistical significance. **Figure S3.** *DLL1* Pyrosequencing results by individual CpG position. Three CpG sites corresponding to the H3K27ac/H3K4me1 peaks were assayed by pyrosequencing on the same samples analyzed by MethylC-seq (region shown by gray bar in Fig. 4a within circled HMD). (PDF 3999 kb)

## Abbreviations

ADOS: Autism Diagnostic Observation Schedule
ASD: Autism spectrum disorder
ChromHMM: Hidden Markov models of chromatin states
CNV: Copy number variation
FDR: False discovery rate
HMD: Highly methylated domain
MARBLES: Markers of Autism Risk in Babies, Learning Early Signs
MethylC-seq: Whole genome bisulfite sequencing
PMD: Partially methylated domain
StochHMM: Hidden Markov models of methylation
TD: Typically developing
