# Supplementary material for: Placental methylome analysis from a prospective autism study
Source: Mol Autism. 2016 Dec 15;7:51. doi: 10.1186/s13229-016-0114-8 (PMC5159983; doi:10.1186/s13229-016-0114-8)
Supplement: Additional file 2: Figures S1-S3. — Figure 1. Histograms of average methylation in 20 kb windows for each placental sample. Individual histograms (no smoothing) for each MARBLES typical and autism placentas as well as three biological replicates of “normal” non-MARBLES placentas which have no known pathologies [16]. Methylation data was omitted if a window had fewer than 20 covered CpG sites. Red vertical lines show the arbitrary 60% methylation cutoff between windows with low and high methylation. Percentage of 20 kb windows with average methylation below 60% also shown in red (excluding omitted windows). n = number of windows with required CpG coverage, m = number of windows omitted due to insufficient coverage. Figure S2. Plots showing location and statistical significance of differential methylation in HMDs and PMDs. Each point shows the FDR-corrected probability of that HMD/PMD being differentially methylated between autism and typical MARBLES placentas. Chromosomes are alternately colored red and black for clarity. The blue line shows the 0.05 cutoff for statistical significance. Figure S3. DLL1 Pyrosequencing results by individual CpG position. Three CpG sites corresponding to the H3K27ac/H3K4me1 peaks were assayed by pyrosequencing on the same samples analyzed by MethylC-seq (region shown by gray bar in Fig. 4a within circled HMD). (PDF 3999 kb) [file 13229_2016_114_MOESM2_ESM.pdf]

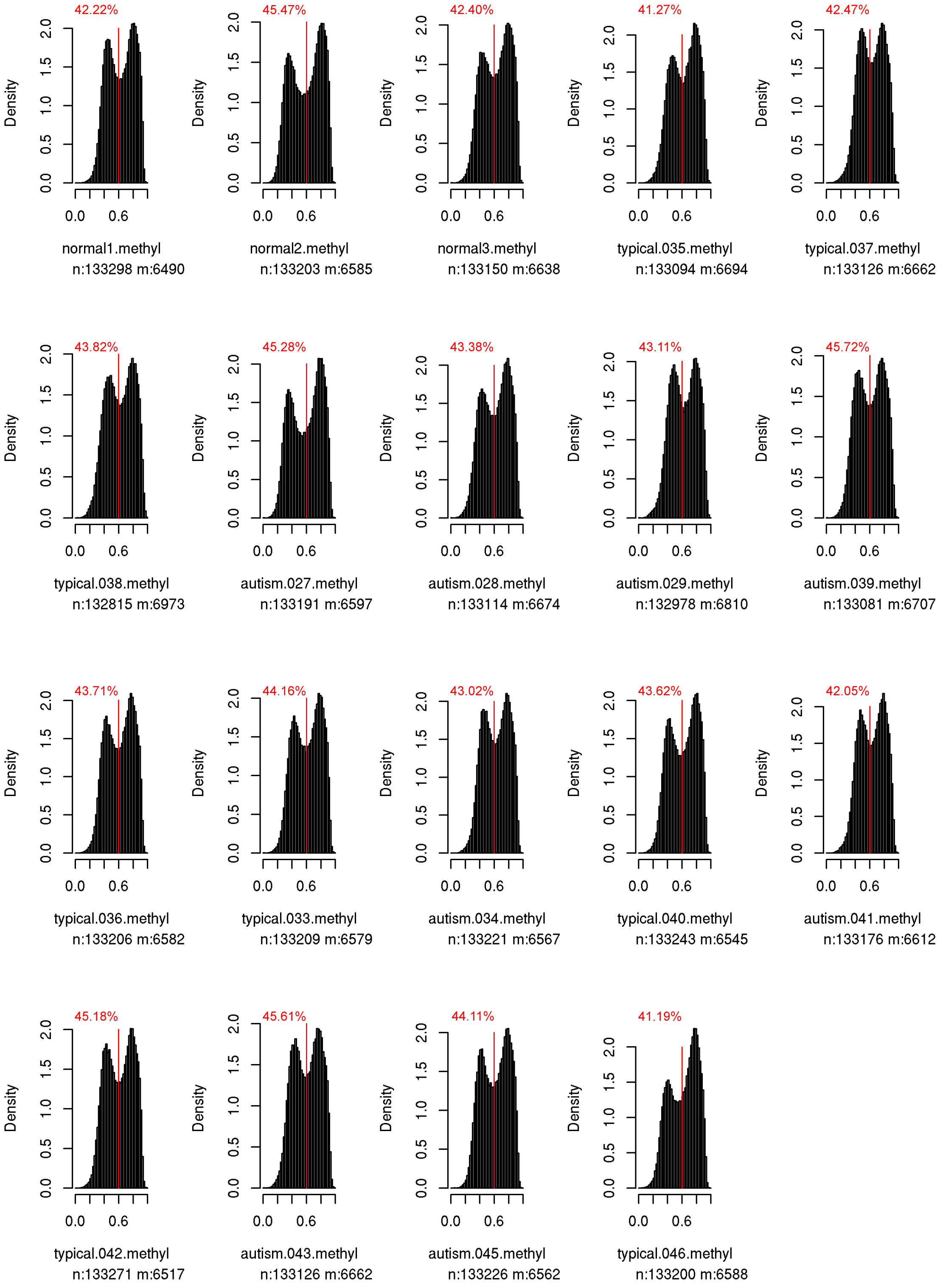
42.22%
normal1.methyl
n:133298 m:6490
45.47%
normal2.methyl
n:133203 m:6585
42.40%
normal3.methyl
n:133150 m:6638
41.27%
typical.035.methyl
n:133094 m:6694
42.47%
typical.037.methyl
n:133126 m:6662
43.82%
typical.038.methyl
n:132815 m:6973
45.28%
autism.027.methyl
n:133191 m:6597
43.38%
autism.028.methyl
n:133114 m:6674
43.11%
autism.029.methyl
n:132978 m:6810
45.72%
autism.039.methyl
n:133081 m:6707
43.71%
typical.036.methyl
n:133206 m:6582
44.16%
typical.033.methyl
n:133209 m:6579
43.02%
autism.034.methyl
n:133221 m:6567
43.62%
typical.040.methyl
n:133243 m:6545
42.05%
autism.041.methyl
n:133176 m:6612
45.18%
typical.042.methyl
n:133271 m:6517
45.61%
autism.043.methyl
n:133126 m:6662
44.11%
autism.045.methyl
n:133226 m:6562
41.19%
typical.046.methyl
n:133200 m:6588
Density
0.0
0.5
1.0
1.5
2.0
0.6

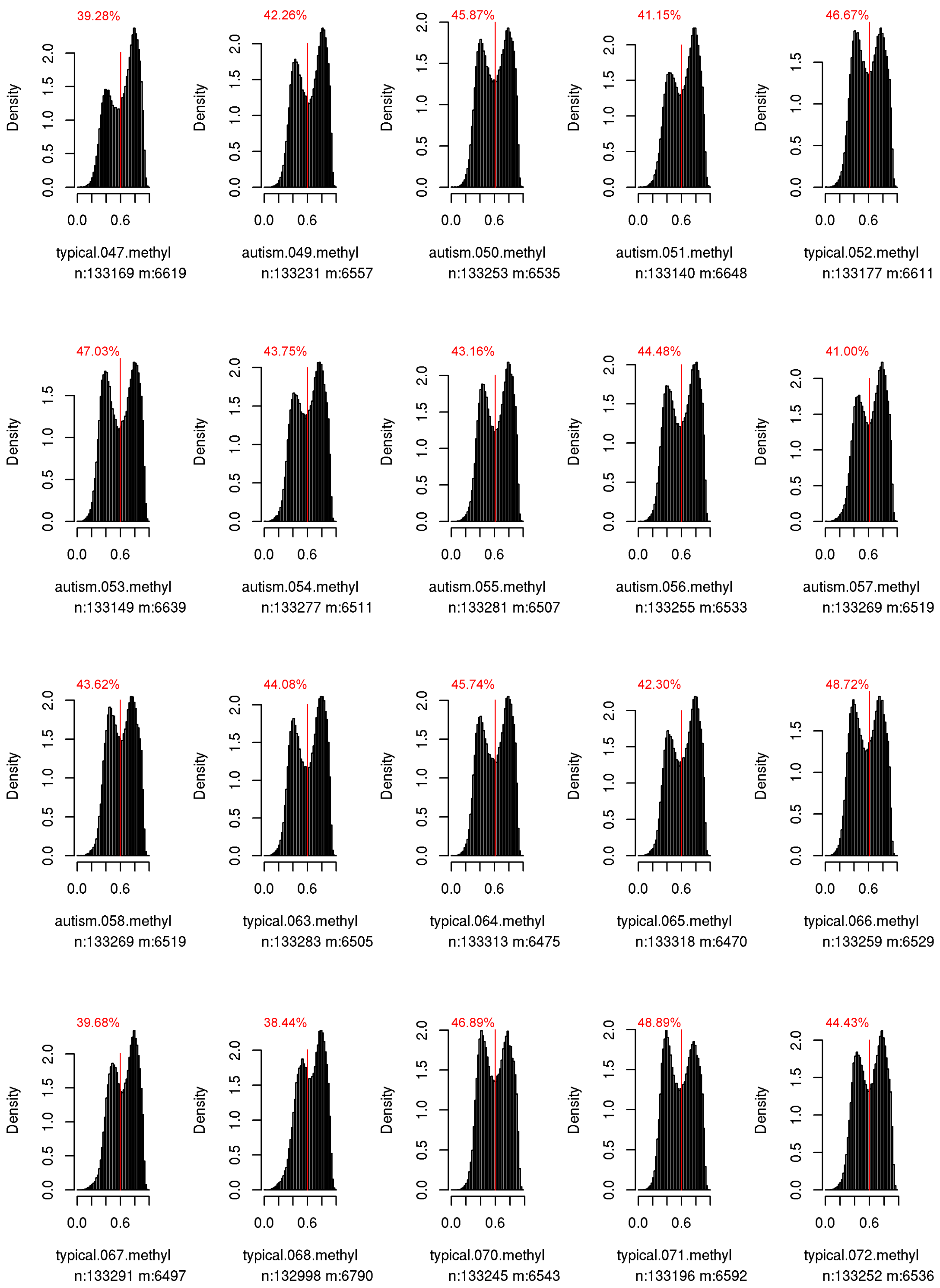
39.28%
typical.047.methyl
n:133169 m:6619
42.26%
autism.049.methyl
n:133231 m:6557
45.87%
autism.050.methyl
n:133253 m:6535
41.15%
autism.051.methyl
n:133140 m:6648
46.67%
typical.052.methyl
n:133177 m:6611
47.03%
autism.053.methyl
n:133149 m:6639
43.75%
autism.054.methyl
n:133277 m:6511
43.16%
autism.055.methyl
n:133281 m:6507
44.48%
autism.056.methyl
n:133255 m:6533
41.00%
autism.057.methyl
n:133269 m:6519
43.62%
autism.058.methyl
n:133269 m:6519
44.08%
typical.063.methyl
n:133283 m:6505
45.74%
typical.064.methyl
n:133313 m:6475
42.30%
typical.065.methyl
n:133318 m:6470
48.72%
typical.066.methyl
n:133259 m:6529
39.68%
typical.067.methyl
n:133291 m:6497
38.44%
typical.068.methyl
n:132998 m:6790
46.89%
typical.070.methyl
n:133245 m:6543
48.89%
typical.071.methyl
n:133196 m:6592
44.43%
typical.072.methyl
n:133252 m:6536
Density
0.0
0.5
1.0
1.5
2.0
0.6

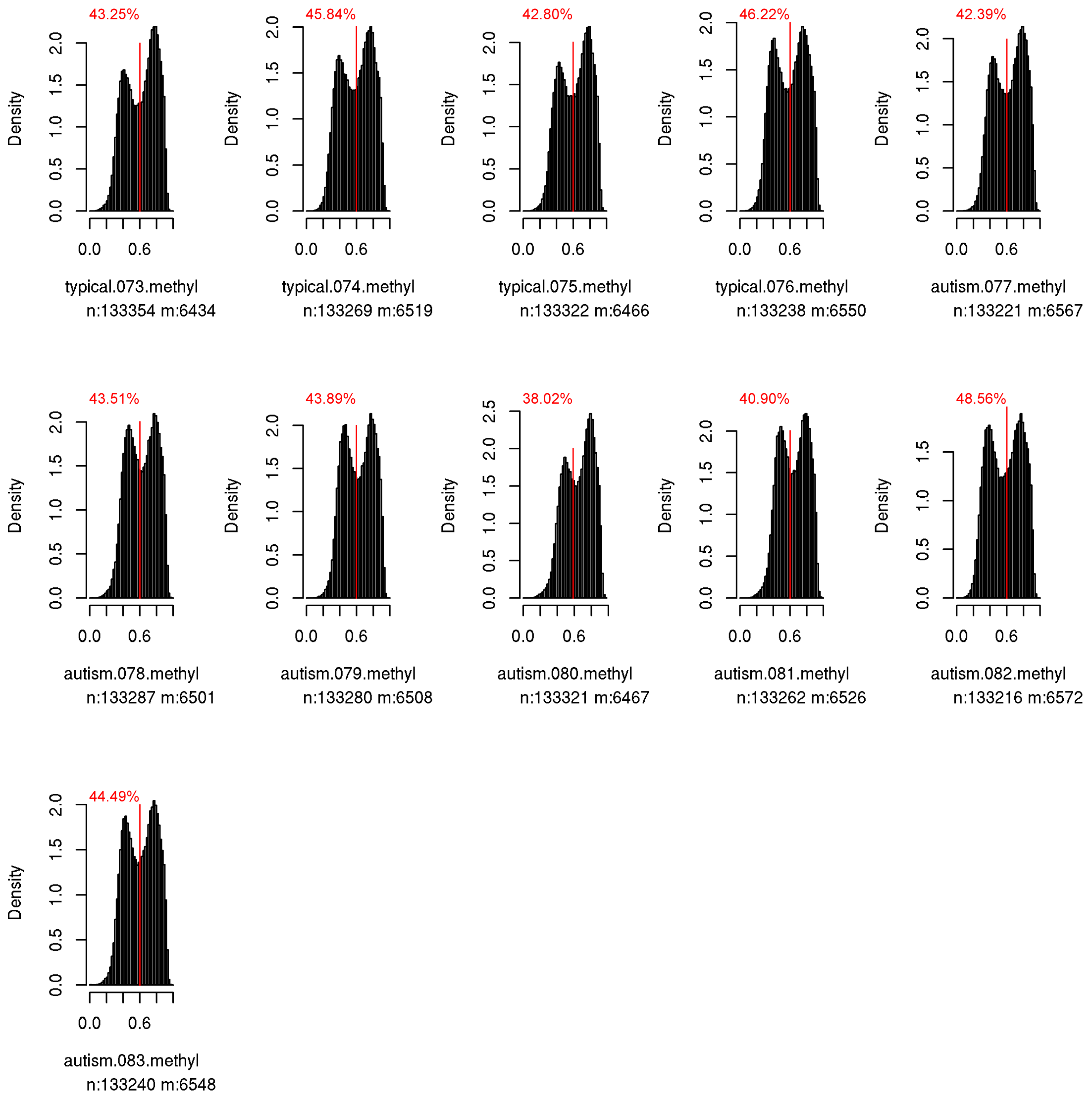

Figures S1: Histograms of average methylation in 20 kb windows for each placenta sample.

Individual histograms (no smoothing) for each MARBLES typical and autism placenta as well as three biological replicates of "normal" non-MARBLES placentas which no known pathologies (Schroeder DI et al., 2013). Methylation data was omitted if a window had fewer than 20 covered CpG sites. Red vertical lines show the arbitrary 60% methylation cutoff between windows with low and high methylation. Percentage of 20 kb windows with average methylation below 60% also shown in red (excluding omitted windows). n=number of windows with required CpG coverage, m=number of windows omitted due to insufficient coverage

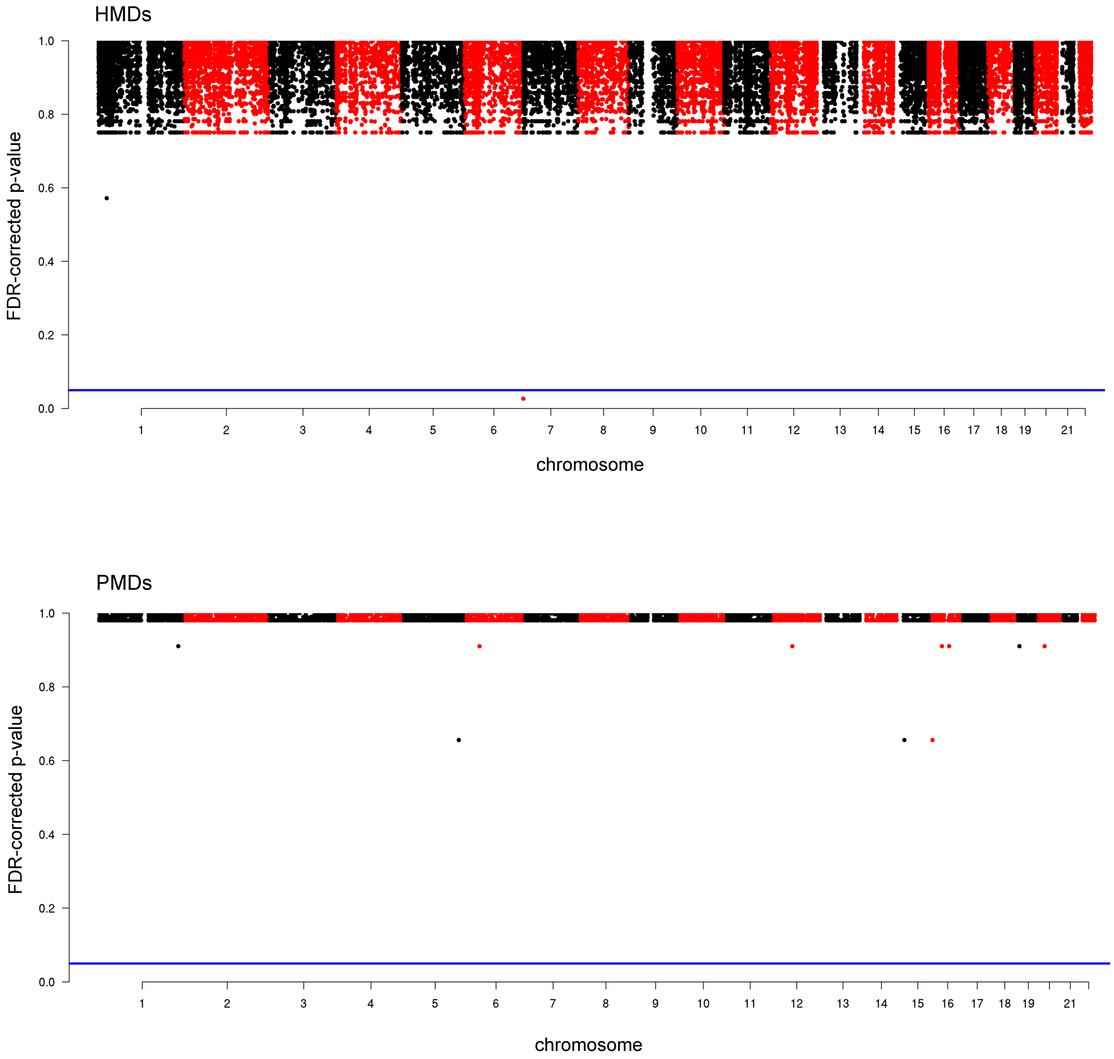

Figure S2: Plots showing location and statistical significance of differential methylation in HMDs and PMDs.

Each point shows the FDR-corrected probability of that HMD/PMD being differentially methylated between autism and typical MARBLES placentas. Chromosomes are alternately colored red and black for clarity. The blue line shows the 0.05 cutoff for statistical significance.

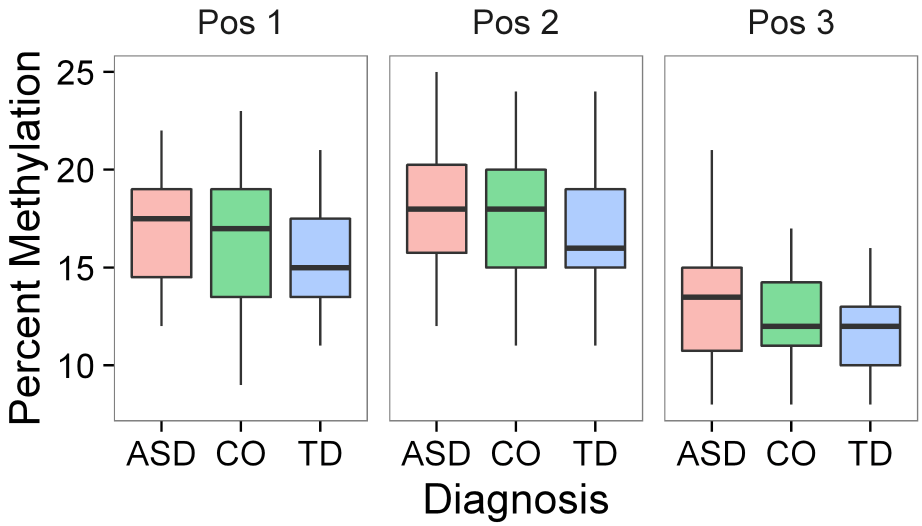

Figure S3: *DLL1* Pyrosequencing results by individual CpG position. Three CpG sites corresponding to the H3K27ac/H3K4me1 peaks were assayed by pyrosequencing on the same samples analyzed by MethylC-seq (region shown by grey bar in Figure 4A within circled HMD).
